# Model Selection for Cogitative Diagnostic Analysis of the Reading Comprehension Test

**DOI:** 10.3389/fpsyg.2021.644764

**Published:** 2021-08-13

**Authors:** Hui Liu, Yufang Bian

**Affiliations:** ^1^Faculty of Linguistic Sciences, Beijing Language and Culture University, Beijing, China; ^2^Collaborative Innovation Center of Assessment for Basic Education Quality, Beijing Normal University, Beijing, China

**Keywords:** continuous variable, diagnostic study, multidimensional item response theory, model selection, reading comprehension test

## Abstract

Reading subskills are generally regarded as continuous variables, while most models used in the previous reading diagnoses have the hypothesis that the latent variables are dichotomous. Considering that the multidimensional item response theory (MIRT) model has continuous latent variables and can be used for diagnostic purposes, this study compared the performances of MIRT with two representatives of traditionally widely used models in reading diagnoses [reduced reparametrized unified model (R-RUM) and generalized deterministic, noisy, and gate (G-DINA)]. The comparison was carried out with both empirical and simulated data. First, model-data fit indices were used to evaluate whether MIRT was more appropriate than R-RUM and G-DINA with real data. Then, with the simulated data, relations between the estimated scores from MIRT, R-RUM, and G-DINA and the true scores were compared to examine whether the true abilities were well-represented, correct classification rates under different research conditions for MIRT, R-RUM, and G-DINA were calculated to examine the person parameter recovery, and the frequency distributions of subskill mastery probability were also compared to show the deviation of the estimated subskill mastery probabilities from the true values in the general value distribution. The MIRT obtained better model-data fit, gained estimated scores being a more reasonable representation for the true abilities, had an advantage on correct classification rates, and showed less deviation from the true values in frequency distributions of subskill mastery probabilities, which means it can produce more accurate diagnostic information about the reading abilities of the test-takers. Considering that more accurate diagnostic information has greater guiding value for the remedial teaching and learning, and in reading diagnoses, the score interpretation will be more reasonable with the MIRT model, this study recommended MIRT as a new methodology for future reading diagnostic analyses.

## Introduction

In the area of language testing, it is reasonable to expect diagnostic information because any language assessment actually has the potential to provide some diagnostic information (Bachman, 1990; Mousavi, 2002), and indeed, there is a series of reading diagnostic studies that have successfully been conducted. However, in the previous reading diagnoses, little discussion on the continuous nature of reading subskills has been provided, though reading subskills are generally regarded as continuous variables (Griffin and Nix, 1991; Lumley, 1993; Grosjean, 2001; Smith, 2004). Although Buck and Tatsuoka (1998) noted that dichotomizing continuous variables are a problem of their diagnostic study, diagnostic studies focusing on the continuous nature of reading comprehension test data have remained elusive. Indeed, when previous reading diagnostic studies have been performed, scarce diagnostic models could have addressed the continuous latent variables, and this lack of diagnostic models for continuous latent variables has been partially responsible for dichotomizing continuous variables under the methodological requirements in previous studies. Model selection is highly important for data analyses (Burnham and Anderson, 2002), and reading diagnostic analysis is no exception. The potential to provide abundant diagnostic information about the reading ability of each test-taker is what makes reading diagnoses so attractive to teachers, administrators, and other language educators who are concerned with teaching and learning of reading, whereas, without a proper model, it is very difficult to make accurate inferences about weaknesses and strengths of test-takers, let alone to make the reading diagnosis truly become the interface between learning and assessment (Alderson, 2005; Kunnan and Jang, 2009). Reading comprehension test data consisting of continuous latent variables are the target data of this present study, and we investigate the performances of different models when they are used in reading diagnoses, aiming to determine whether a diagnostic model with continuous latent variables will gain an advantage when used in reading diagnostic analyses.

### Reading Diagnostic Studies: Current Status and Issues

The existence of distinguishable reading subskills (Grabe, 2009; Bernhardt, 2011) implies the multidimensional nature of reading ability, which renders the diagnostic score report possible. The first batch of reading diagnostic studies is based on the rule-space model (RSM), and the study by Buck et al. (1997) is representative of this line of research. Buck et al. (1997) analyzed the reading scores of 5,000 Japanese students on the Test of English for International Communication (TOEIC), classified 91% of the test-takers into their latent knowledge states, and provided them with diagnostic information based on a set of attributes. The study by Jang (2009) is typical of more recent research. In the context of *LanguEdge*^™^ test items, Jang suggested that the fusion model with all C parameters being set to 10 [i.e., the reduced reparametrized unified model, R-RUM, which was discussed in detail by Jang (2005)] is appropriate and can provide test-takers with more fine-grained diagnostic information regarding their reading abilities. Readers interested in additional details about previous reading diagnostic studies are referred to by Lee and Sawaki (2009b) and Li et al. (2016). Meanwhile, we also observe limitations in these reading diagnostic studies: the chief models contain only dichotomous (mastery vs. non-mastery) subskills.

Regarding the subskill scale, the cognitively diagnostic psychometric model has two categories, which are the continuous scale and the discrete (i.e., dichotomous or polytomous) scale (Fu and Li, 2007). The major distinction between them is the type of the latent variables used: continuous latent variables are used in the former, whereas discrete latent variables are used in the latter (Rupp et al., 2010). Researchers have emphasized that whether the variable in question is continuous or discrete determines which type of model should be chosen for the actual application (Stout, 2007). Specific to reading comprehension tests, the variable in question is the reading subskill.

A continuous variable is a variable that can take on any value in its possible range, which is in opposition to a discrete variable (Mackey and Gass, 2005). Although a consensus is lacking, many linguists believe that reading develops gradually and that the reading subskills of individuals are at different points on a continuous scale (Griffin and Nix, 1991; Lumley, 1993; Grosjean, 2001; Smith, 2004). Moreover, on reviewing the subskills specified in previous reading diagnostic studies, it is found that subskills are continuous in opposition to discrete latent variables; regardless, the grain size of a subskill is larger (Alderson, 2005; Lee and Sawaki, 2009b) or smaller (Jang, 2009). The continuous nature of reading subskills implies that the continuous scale model is theoretically more appropriate in the context of reading comprehension test data.

The literature review, however, reveals that dichotomous scale models are the traditional choice in reading diagnoses and that virtually all previous reading diagnostic studies have used dichotomous scale models (Lee and Sawaki, 2009b; Li et al., 2016). Some problems may exist with this traditional choice because, in general, statistical models should be used only for data that meet their theoretical assumptions; moreover, it is acknowledged that difficulty will arise in the score interpretation of a reading diagnosis under the framework of a dichotomous subskill scale (Jang, 2009). At present, continuous scale models that are able to conduct diagnostic analysis are available. The multidimensional item response theory (MIRT) model is one of the most popular diagnostic models with a continuous scale (Reckase, 2009), and it has already been used to report subskill scores (Yao and Boughton, 2007; Haberman and Sinharay, 2010). Considering the continuous nature of reading subskills, the MIRT is expected to have an advantage in extracting diagnostic information from reading comprehension tests.

### Representative Models

Comparison studies have been conducted on the popular models in reading diagnoses. Lee and Sawaki (2009a) compared the functioning of three diagnostic models, i.e., the general diagnostic model (GDM), the reparametrized unified model (RUM), and the latent class analysis, when used to analyze the reading and listening sections of the TOEFL iBT. Li et al. (2016) to compare the performances of five models, namely, the generalized deterministic inputs, noisy “and” gate (G-DINA), additive cognitive diagnostic model (ACDM), the R-RUM, and the DINA and DINO models, according to their applicability to the Michigan English Language Assessment Battery (MELAB) reading test.

Overall, the R-RUM has received a fairly intensive study and shown good performances in previous research (Jang, 2009; Lee and Sawaki, 2009b), and Li et al. (2016) showed that G-DINA is better fitted for use in a reading diagnostic study. Consequently, in this study, the R-RUM and G-DINA are selected as representatives of the traditionally widely used models for comparison with the MIRT model. For the sake of brevity, the introduction of the three models is relegated to the Appendix (Supplementary Material).

In addition, it is necessary to indicate that, in this study, the MIRT model considered is the compensatory MIRT model. The two basic types of MIRT include the compensatory and non-compensatory models. Bolt and Lall (2003) suggested that the compensatory MIRT model fits the real data from an English usage test better than the non-compensatory MIRT and fits the data generated from the non-compensatory model nearly as well as the non-compensatory model itself. Given the comparison results of Bolt and Lall (2003) and the suggestion of many current language researchers that reading subskills are generally compensatory (Stanovich, 1980; Grabe, 2009), the MIRT model considered in this study is the compensatory MIRT model. In addition, MIRT analyses can be either exploratory or confirmatory, and when used in diagnostic analyses, the model should be confirmatory (Reckase, 2009), because it requires a hypothesis for the structure of the response data, which is identical to the Q-matrix in dichotomous subskill scale models.

As noted above, there have been continuous scale diagnostic models. Given the diagnostic potential of the MIRT model (Stout, 2007) and its capacity to be fitted to continuous latent variables, we expect to determine whether the MIRT model is more appropriate than the traditionally widely used models in reading diagnoses. To that end, the performances of MIRT and representatives of the traditionally widely used models (R-RUM and G-DINA) are examined with both real and simulated data based on a reading comprehension test. The first aim of this study is to examine whether the MIRT model has a better model-data fit. Second, we emphasize assessing the extent to which the estimated subskill scores represent the true abilities of the test-takers. Third, we put emphasis on assessing the person parameter recovery with the correct classification rates. Finally, this study is undertaken to compare the three candidate models deviations from the true values on the frequency distribution of subskill mastery probabilities. We believe that this comparison will provide insight into model selection for cogitative diagnostic analyses of reading comprehension tests.

## Materials and Methods

The reduced reparametrized unified model, G-DINA, and MIRT were used to calibrate real and simulated data based on a reading comprehension test, and their performances were compared in this study. Different indices, such as the Akaike Information Criterion (AIC) and Bayesian Information Criterion (BIC) can be used to evaluate the model-data fit in a model comparison (Ma et al., 2016); thus, in this study, first, these relative model-data fit indices were compared across the three models with real data and the absolute model-data fit indices were also provided to assess whether the chosen model really fits the real data well. Then, further analyses were conducted with simulated data. Because the “true” values are known in simulation studies, the deviation of the estimated values from “true” values is comparable. With simulated data, the functioning of the three models was examined in terms of the extent to which the true abilities were represented and the correct classification rates under different research conditions, and the corresponding frequency distributions of the subskill mastery probabilities were also compared.

### Real Data

Response data from 3,077 students on 30 reading comprehension items were collected from a large-scale Grade 5 and 6 reading comprehension test in Beijing, P.R. China. The test is an existing reading comprehension test that was not designed to be diagnostic, and the Q-matrix in Table 1, which was built and validated through the literature review and think-aloud protocols, displays the correspondence between the three subskills and 30 items.

**Table 1:** Q-matrix for this study.

**Item**	**Subskill 1** **(retrieving** **information)**	**Subskill 2** **(making straightforward** **inference)**	**Subskill 3** **(making integrated** **inference)**
1	1	0	0
2	1	1	0
3	0	1	0
4	1	1	0
5	1	1	0
6	0	0	1
7	1	0	0
8	1	0	0
9	1	1	0
10	0	1	0
11	0	1	0
12	1	1	0
13	1	0	0
14	1	0	0
15	1	1	0
16	0	1	0
17	1	1	0
18	0	0	1
19	1	1	0
20	1	0	0
21	0	0	1
22	0	0	1
23	0	0	1
24	0	0	1
25	0	0	1
26	0	0	1
27	0	0	1
28	0	1	1
29	0	1	0
30	0	0	1

### Simulation Study

We simulated response data based on the real data calibration with the MIRT model, which is a typical choice when multidimensional continuous latent variables are desired.

#### Item Parameters

The item parameters used to generate the simulated data were obtained from the real data with a three-dimensional MIRT calibration estimated by the flexMIRT software program (Cai, 2015). Table 2 shows the true item parameters. The first column lists the item number, the second to fourth columns present the three discrimination parameters for subskill 1, subskill 2, and subskill 3, respectively, and the last two columns show the intercept and guessing parameters.

**Table 2:** True item parameters.

**Item**	**Discrimination**			**d**	**c**
	**a_**1**_**	**a_**2**_**	**a_**3**_**		
1	1.10	0.00	0.00	2.51	0.23
2	0.61	0.72	0.00	0.54	0.17
3	0.00	0.98	0.00	1.12	0.15
4	0.85	0.77	0.00	0.49	0.29
5	0.70	0.58	0.00	−0.42	0.10
6	0.00	0.00	1.30	−0.53	0.56
7	1.38	0.00	0.00	0.48	0.53
8	1.32	0.00	0.00	−0.15	0.44
9	1.05	1.02	0.00	2.09	0.34
10	0.00	1.34	0.00	0.93	0.28
11	0.00	0.90	0.00	0.87	0.21
12	0.68	0.85	0.00	2.54	0.38
13	1.33	0.00	0.00	2.73	0.13
14	1.32	0.00	0.00	1.44	0.14
15	1.40	1.08	0.00	1.10	0.32
16	0.00	2.03	0.00	4.89	0.17
17	1.08	1.55	0.00	5.01	0.15
18	0.00	0.00	0.70	1.95	0.23
19	0.56	0.56	0.00	2.58	0.22
20	1.40	0.00	0.00	5.01	0.23
21	0.00	0.00	0.98	1.79	0.20
22	0.00	0.00	0.87	1.44	0.12
23	0.00	0.00	1.38	2.33	0.19
24	0.00	0.00	1.52	2.43	0.18
25	0.00	0.00	0.50	−0.30	0.16
26	0.00	0.00	1.60	3.17	0.17
27	0.00	0.00	1.07	0.94	0.07
28	0.00	0.93	1.04	1.10	0.23
29	0.00	0.53	0.00	0.23	0.23
30	0.00	0.00	0.69	0.58	0.13

*Item d is a scalar parameter related to the difficulty of the corresponding item*.

#### Simulation Conditions

The abilities for 3,000 test-takers were generated from multinormal distributions with the mean of (0, 0, 0) and σ as the variance-covariance matrix, and the simulated response datasets were produced with these abilities, in which:

$$\sigma =\left(\begin{array}{ccc} a_{1} & r_{1} & r_{2}\\ r_{1} & a_{2} & r_{3}\\ r_{2} & r_{3} & a_{3} \end{array}\right)$$

where, a_1_ = a_2_ = a_3_ = 1 and r_1_ = r_2_ = r_3_ = 0.1, 0.3, 0.5, 0.7, and 0.9.

The manipulated design factor for the simulated data generation was the subskill correlations (SCs). Considering that their correlations can vary over a relatively wide range, from a negligibly small value (Guthrie and Kirsch, 1987) to a moderately and a relatively high relationship between reading subskills (Droop and Verhoeven, 2003; Alderson, 2005), the SCs were found to be 0.1, 0.3, 0.5, 0.7, and 0.9, as noted above, and the correlations between the subskills were set equal to reduce the simulation conditions to be studied (Yao and Boughton, 2007).

Therefore, we had five simulation conditions. Twenty-five different seeds were used to obtain 25 replications across all simulation conditions; thus, there were 125 simulated datasets in total.

#### Subskillability Estimates and Classification Procedures

For each simulated dataset, three calibrations using R-RUM, G-DINA, and MIRT were performed. The R-package CDM was used to estimate the R-RUM and G-DINA model, and flexMIRT was used to estimate the MIRT model. R-RUM and G-DINA report the MLE and MAP estimates of subskill mastery patterns to each test-taker, and MLE estimates were used in this study; they also provide the posterior mastery probabilities of test-takers on each subskill. The MIRT model provides every test-taker a **θ**-vector that indicates their proficiency on each subskill, which can easily be transformed into vectors of mastery probabilities, given the multinormal nature of the simulated datasets.

In this simulation part of the present study, there should be four subskill scores per test-taker per subskill, i.e., one true subskill score representing the true ability of the test-taker from the simulated true value, and three estimated subskill scores representing the estimated abilities of the test-taker from R-RUM, G-DINA, and MIRT. The true subskill score and the estimated subskill score from MIRT are the *θ* abilities on each subskill, which would fall along a continuous subskill scale, and the estimated subskill scores from R-RUM and G-DINA indicate the classification result of “mastery” or “non-mastery,” termed the mastery state, which would fall along a dichotomous subskill scale. In this study, the extent to which the estimated subskill scores represent the true abilities of the test-takers were evaluated through comparison of relations between the estimated scores from MIRT, R-RUM, and G-DINA and the true scores.

Furthermore, in the diagnostic study context, the classification results are expected. Classifications of test-takers are the bases for providing remedial strategies to facilitate learning, so whether the remedial strategies provided for improvement are effective depend mainly on the accuracy of the classification results. Therefore, it is also very important for the diagnostic model selection to evaluate the extent to which the approximating models classify the test-takers into their intended groups. As mentioned above, we classify the performances of the test-takers on a subskill into different groups, such as the “mastery” and “non-mastery” group, and the classification result is termed the mastery state. In this simulation study, there should be four classification results per test-taker per subskill, i.e., one true mastery state from the simulated true value, and three estimated mastery states from R-RUM, G-DINA, and MIRT. We know that the estimated mastery states from R-RUM and G-DINA can be obtained directly, while true mastery states of the test-takers and their estimated mastery states from MIRT cannot be obtained unless we dichotomize each continuous *θ* scale with a cut-off point (American Educational Research Association, American Psychological Association, and National Council on Measurement in Education, 2014). There is no natural cut-off points for a continuous variable; in addition, the establishment of a cut-off point should be based on a labor-intensive and time-consuming process (Hambleton and Pitoniak, 2006), and the implementation of this process is beyond the scope of this present study. Therefore, in this simulation study, different cut-off points were used to cover different practical needs: we set the cut-off point to increase from 0.1 to 0.9 with a step of 0.01 on the subskill mastery probability scale transformed from the *θ* scale, considering that a cut-off point is often defined in the form of a value established on the subskill mastery probability scale in diagnostic analyses (DiBello and Stout, 2008). In this way, we obtained the true subskill mastery state and the estimated subskill mastery state from the MIRT with each of the 81 cut-off points. Consequently, the comparison of classification results can be conducted. For example, if on subskill 1, the true mastery probability of test-taker A is 0.11, the estimated mastery states from R-RUM and G-DINA are “non-mastery” and “mastery,” and the estimated mastery probability from MIRT is 0.15, then with a cut-off point of 0.1, the true mastery state of test-taker A on subskill 1 is “mastery,” and the estimated mastery states of test-taker A to this subskill from R-RUM, G-DINA, and MIRT are “non-mastery,” “mastery,” and “mastery,” which indicate the successful estimations of G-DINA and MIRT and of the mastery of subskill 1 of test-taker A; while with a cut-off point of 0.12, only R-RUM would correctly estimate the non-mastery of subskill 1 of test-taker A.

We have five simulation conditions, i.e., the five subskill correlations used in generating simulated data, and 81 cut-off points are used to dichotomize each *θ* scale in each simulated dataset, producing 405 (5 × 81) research conditions in total.

#### Evaluation Criteria

In the simulation section, the deviation of the estimated values from true values was compared in three ways. First, we compared the relations between the estimated subskill scores and the true subskill score, with the former containing both the estimated mastery states on each subskill reported in the subskill mastery patterns from the two dichotomous scale models and the estimated *θ* values on each subskill from MIRT. Second, the subskill classification accuracies were compared, and the indices are described in detail in the Results section. Finally, the estimated frequency distributions of the subskill mastery probabilities were compared to the true distribution too.

When comparing the subskill classification accuracies, previous studies always use the correct classification rates to evaluate the classification consistency between the true values and the estimated values (Ma et al., 2016), which involves the pattern correct classification rate (PCCR) and the subskill correct classification rate (SCCR) in this study. PCCR and SCCR are defined as,

(1) $$\begin{array}{c} PCCR=\frac{\sum_{r=1}^{Rep}\sum_{i=1}^{N}t_{i}}{N\times Rep}, \end{array}$$

where *PCCR* is the index used to evaluate the classification recovery accuracy of the subskill mastery pattern, which refers to a vector that involves the mastery states of a test-taker on all subskills; N is the total number of test-takers; Rep is the number of replications; and *t*_*i*_ indicates whether the estimated mastery pattern of a test-taker *i* is the same as the true pattern, with a value of 1 if the two are identical and a value of 0, if not.

(2) $$SCCR=\frac{\sum_{r=1}^{Rep}\sum_{i=1}^{N}\sum_{k=1}^{k}g_{ik}}{N\times K\times Rep},$$

Where, *SCCR*_*k*_ is the index used to evaluate the classification recovery accuracy of subskill *k*, and *g*_*ik*_ indicates whether the estimated mastery state of test-taker, *i* on subskill, *k* is the same as the true state, with a value of 1 if the two are identical, and a value of 0, if not.

In the present study, PCCRs and SCCRs were compared among R-RUM, G-DINA, and MIRT across all research conditions.

## Results

### Model-Data Fit With Real Data

The real data are calibrated using the three models, i.e., R-RUM and G-DINA with R-package CDM, and MIRT with flexMIRT, and the estimations are based on the EM algorithm with the default data (de la Torre, 2011; Cai, 2015; Robitzsch et al., 2015). Indices, such as the AIC and BIC are used to evaluate the model-data fit and to aid in the model comparison (Henson et al., 2009), which is also used in the model selection for reading diagnoses (Li et al., 2016). These goodness-of-fit (GOF) indices are reported by both R-package CDM and flexMIRT and are used for model selection. A summary of these model-data fit indices is presented in Table 3, which contains the −2 × log-likelihood, AIC, and BIC. The smaller values of these indices tend to accept the null hypothesis, which indicates a good model-data fit.

**Table 3:** Summary of goodness-of-fit statistics of the three models.

**Index**	**R-RUM**	**G-DINA**	**MIRT**
Number of parameters	76	85	99
−2 × log-likelihood	79,805.49	79,774.96	79,393.43
AIC	79,957.49	79,944.96	79,591.43
BIC	80,415.90	80,457.65	80,188.57

As shown in Table 3, the GOF statistics are strikingly different between the MIRT model and the two representatives of the traditionally widely used models, where MIRT has the smallest value in all three indices. Although G-DINA and R-RUM have similar performances on these GOF statistics, the G-DINA model has a smaller −2 × log-likelihood and AIC values and R-RUM has a smaller BIC value. Given that the AIC performed better in choosing diagnostic models (Li et al., 2016) and the BIC always has a strong penalty for highly parameterized models, G-DINA is regarded as a model that fits the real data better than R-RUM.

Considering the −2 × log-likelihood, since AIC and BIC are all relative model-data fit statistics, absolute fit indices of MIRT are required to evaluate whether it really fits data well-absolutely: *M*_2_ and the corresponding root mean square error of approximation (RMSEA) are calculated. *M*_2_ is a limited-information GOF statistics, and it has the benefit of being less sensitive to sparseness than the full-information statistics, such as the Pearson's *X*^2^ or the likelihood ratio *G*^2^ (Cai and Hansen, 2012). A statistic unaffected by the sample size and the model complex degree, the RMSEA ranges from 0 to 1. A smaller RMSEA implies a better GOF, and Browne and Cudeck (1992) recommended 0.05 as the threshold for a *close fit*. In this case, the value of *M*_2_ statistics is 2042.59 (*df* = 366, *P* < 0.0001) and *M*_2_ is rejected. The rejection is not surprising considering that *M*_2_ is sensitive to model misspecification. Fortunately, we have the RMSEA index, which is calculated from *M*_2_ and can be used to evaluate the severity of model misspecification (Cai and Hansen, 2012). The value of the RMSEA is 0.04, which suggests that the MIRT model provides a good fit.

In short, based on the log-likelihood and information criteria, the MIRT model is best fitted to the real reading comprehension test data among the three models, followed by G-DINA and then R-RUM; the advantage of MIRT over the other two models is much greater than the improvement from G-DINA to R-RUM. Based on the *M*_2_ and the RMSEA statistics, we conclude that the MIRT model fits the real data well. Therefore, the GOF indices provide strong evidence for the choice of MIRT.

### Results With Simulated Data

#### Comparison of Subskill Scores

**Figure 1:**
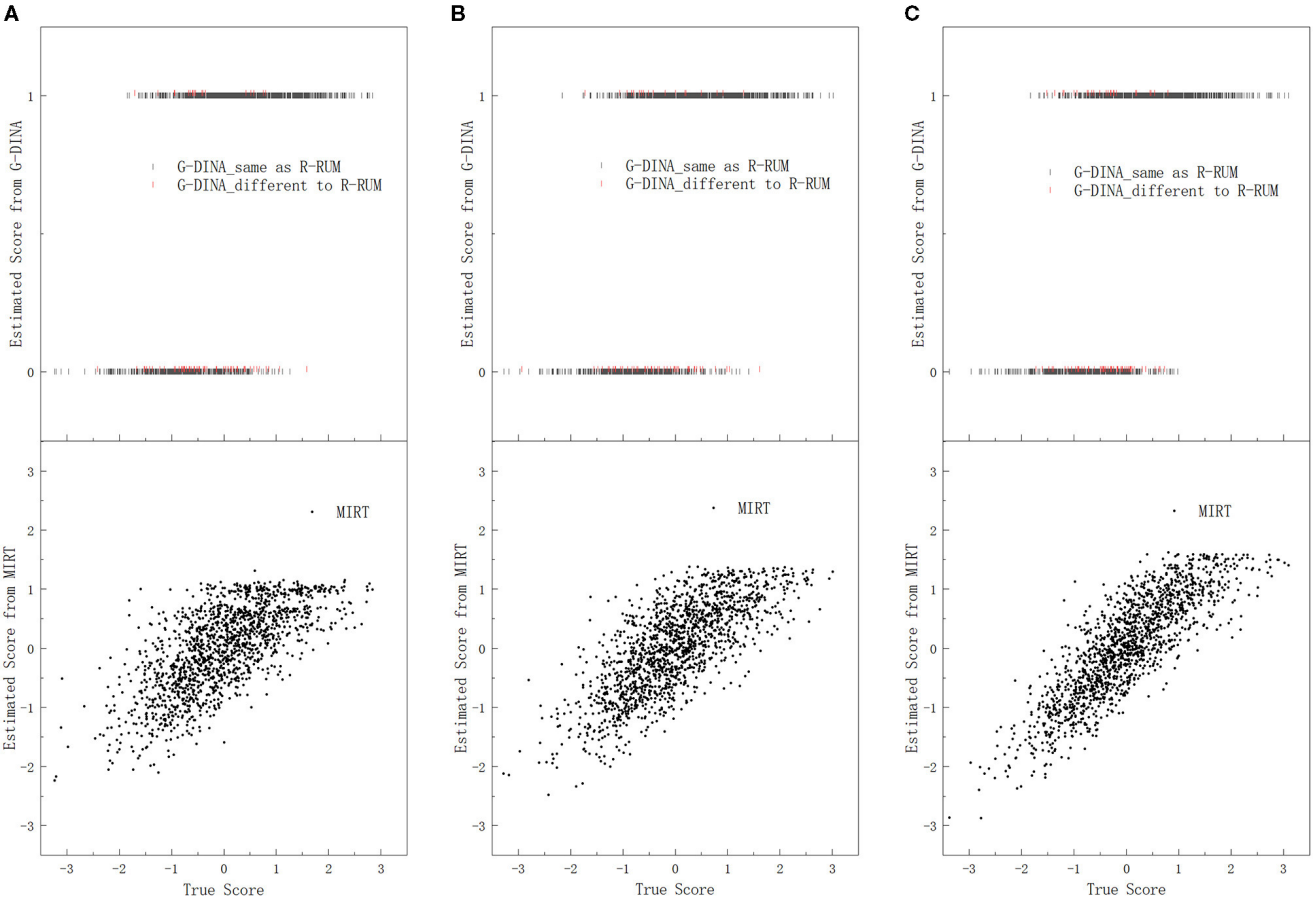
Plots of the estimated subskill scores from the three candidate models against the true subskill scores on subskill 1. **(A)** SC = 0.1, **(B)** SC = 0.5, and **(C)** SC = 0.9.

Under each research condition, we plotted the estimated subskill scores from the three candidate models against the true subskill scores on each subskill, for all the 75,000 (3, 000 × 25) simulated test-takers with every 50th point being plotted. Dozens of scatter plots were produced, because five subskill correlations, three subskills, and three candidate models were used in this study. Figure 1 presents the scatter plots of the estimated subskill scores against the true subskill scores on only subskill 1, as the same pattern was observed for both subskill 2 and subskill 3 conditions. In addition, we excluded the scatter plots under subskill correlations of 0.3 and 0.7 in Figure 1 for the sake of simplicity.

The comparison of the estimated subskill scores and the true subskills scores on subskill 1 under subskill correlations of 0.1, 0.5, and 0.9 is displayed in Figure 1, with a dual *y*-axis coordinate system: the lower *y*-axis represents the estimated subskill scores from MIRT, and the upper *y*-axis denotes the estimated subskill scores from G-DINA. In the upper part of Figure 1, each dark bar (|) represents a pair of scores for one test-taker whose estimated subskill score from G-DINA is the same as that from R-RUM, and each red bar represents that for one test-taker whose estimated subskill score from G-DINA is different to that from R-RUM. Therefore, the relations between the estimated subskill scores from all the three candidate models and their corresponding true values on subskill 1 are shown in Figure 1.

Figure 1 displays the relations between the estimated subskill scores from the two kinds of models and the true values. The lower part of Figure 1 shows that the estimated subskill scores from MIRT tend to increase as the true subskill scores increase, and the correlation between the estimated scores from MIRT and the true scores increases as the correlation between the subskills increases. In addition, the estimated subskill scores from MIRT are approximately centered on their corresponding true subskill scores with estimation bias, and the bias reduces when the subskill correlation is higher. For example, under subskill correlation of 0.1, for all the 305 test-takers with identical true subskill score of 0 (being rounded to two decimal places), their estimated subskill scores from MIRT are centered at 0.08 under normality, with many estimated points being close to the true value.

Regarding the estimated subskill scores from the dichotomous models, R-RUM and G-DINA exhibit similar performances. These two models also have a general tendency that as the true subskill scores increase, the estimated subskill scores tend to rise to score 1 (“mastery”). However, deviation from this tendency clearly exists. The upper part of Figure 1 illustrates that there is a large overlap in the true subskill scores between the estimated groups of mastery and non-mastery, and test-takers whose true subskill scores are located within this overlapping range may be estimated as mastery or non-mastery of subskill 1, no matter whatever be the scores of their true subskill ability. In addition, the estimated scores from the dichotomous models may be totally different for students who have exactly the same true ability. For example, under subskill correlation of 0.9, for the 307 test-takers with identical true subskill score of 0, 220 of them are scored 1 by G-DINA, and 87 are scored 0 by the same model. In brief, Figure 1 indicates that with R-RUM and G-DINA, a large number of the true subskill abilities of the test-takers fall into the broadly overlapping ranges in the true subskill score between the groups of mastery and non-mastery estimated by the two models; the large number of test-takers can be subdivided into many subsets with exactly the same true subskill ability, while test-takers in each subset may obtain totally different estimated scores, and entirely different remedial interventions may be provided to them even though actually they should have identical treatment. This situation implies that the estimated subskill scores from the dichotomous models may not be able to represent the true abilities of the test-takers as well as the estimated subskill scores from MIRT.

#### Correct Classification Rates: SCCR and PCCR

**Figure 2:**
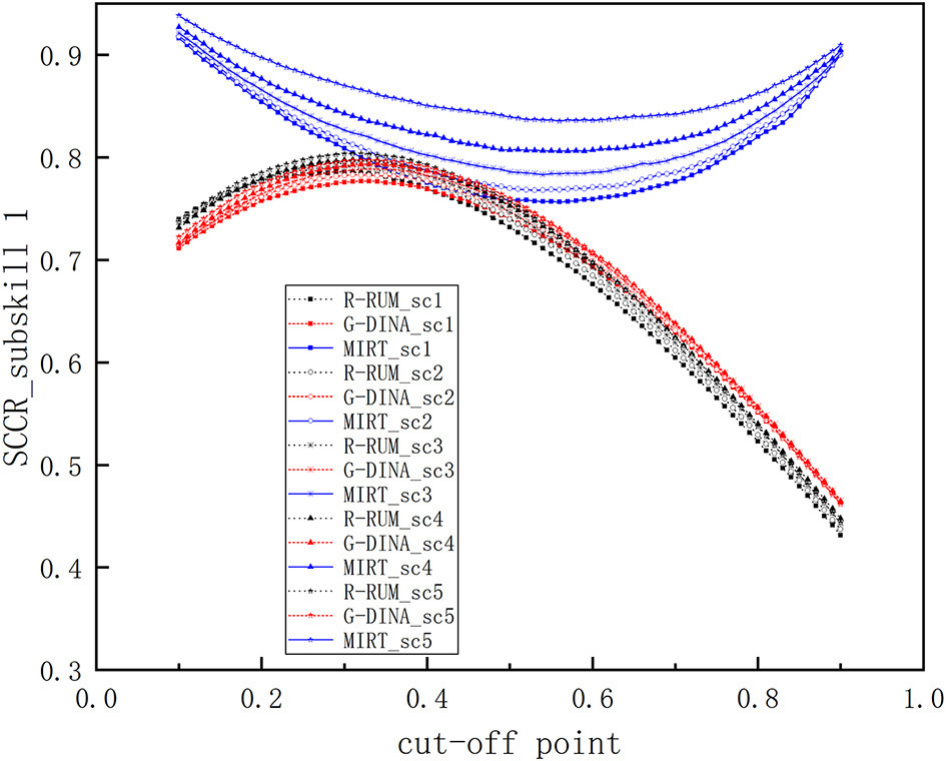
Comparison of *SCCR*_1_s among R-RUM, G-DINA, and MIRT.

Figure 2 illustrates the SCCRs for R-RUM, G-DINA, and MIRT under all research conditions on only subskill 1, as the same pattern was observed for both subskill 2 and subskill 3 conditions. The specific correct classification rate of subskill 1 (*SCCR*_1_) values under different conditions are also given in Table 1A (see Supplementary Material).

Differences in the *SCCR*_1_s clearly exist among the three models. Across the five simulation conditions, the average *SCCR*_1_s includes 0.682–0.699 for R-RUM, 0.688–0.702 for G-DINA, and 0.807–0.865 for MIRT. From Figure 2, we can clearly observe that the *SCCR*_1_s for MIRT are the highest among the three models under all research conditions and that there are fewer differences across the three models when the subskill correlations are lower. For example, when the subskill correlation is 0.1 and the cut-off point is 0.5, the *SCCR*_1_ for MIRT is 0.759, which is higher than the *SCCR*_1_ of 0.732 from R-RUM and the *SCCR*_1_ of 0.740 from G-DINA by 0.027 and 0.019; when the subskill correlation is 0.5, the *SCCR*_1_ for MIRT is also higher than the *SCCR*_1_s from R-RUM and G-DINA, and the MIRT model outperforms R-RUM and G-DINA by 0.040 and 0.033, which is more evident than the improvements of 0.027 and 0.019 when the subskill correlation is 0.1. From Figure 2, we can also observe that there are fewer differences across the three models when the cut-off points are located near positions where the true mastery proportions from the simulated data are similar to the estimated mastery proportions from R-RUM and G-DINA (the estimated mastery proportions from R-RUM and G-DINA are about 0.66 and 0.63, respectively, which are provided in detail in Table 2A in Supplementary Material). For example, when the subskill correlation is 0.3 and the cut-off point is 0.37, the *SCCR*_1_ for MIRT is 0.808, which is higher than the value of 0.789 from R-RUM and the value of 0.787 from G-DINA by 0.019 and 0.021; when the cut-off point is 0.57, the *SCCR*_1_ for MIRT is also higher than the *SCCR*_1_s from R-RUM and G-DINA, and the MIRT model outperforms R-RUM and G-DINA by 0.074 and 0.063, which is more evident than the improvements of 0.019 and 0.021 when the cut-off point is 0.37. In addition, the *SCCR*_1_s for R-RUM and G-DINA are close to each, with R-RUM being slightly better when the subskill correlations are lower, G-DINA being slightly better when the subskill correlations are higher, and the average *SCCR*_1_s over the five simulation conditions for G-DINA being slightly higher.

Regarding the characteristics of the *SCCR*_1_s for the models themselves, it is note that the *SCCR*_1_s increase as the correlations between the subskills increase; for example, when the cut-off point is 0.6, the *SCCR*_1_s for R-RUM increase from 0.676 to 0.698, as the subskill correlations increase from 0.1 to 0.9. The *SCCR*_1_s for the MIRT model are the lowest when the cut-off points depart from near the mean and increase as the cut-off point moves farther away; for R-RUM and G-DINA, their *SCCR*_1_s are the highest when the cut-off points are located near positions, where the true proportions of masters are similar to the estimated proportions of masters from R-RUM and G-DINA, and their *SCCR*_1_s decrease as the cut-off points move farther away. For instance, when the subskill correlation is 0.5, the *SCCR*_1_ of 0.783 is obtained when the cut-off point is 0.54, which is the lowest for MIRT, and *SCCR*_1_ for MIRT increases as the cut-off point moves from 0.54 to 0.1 or 0.9; under the same simulation condition, the *SCCR*_1_ of 0.789 obtained with the cut-off point of 0.34 is the highest for G-DINA and the *SCCR*_1_ for G-DINA decreases as the cut-off point moves farther away from this point.

For the three models, the PCCRs exhibit similar performances to the SCCRs: MIRT holds the highest values under all research conditions and G-DINA is slightly better than R-RUM, the differences among them are smaller when the subskill correlations are lower, and in general, the PCCRs for each single model increase as the correlations between the subskills increase, and so on. Because the general change tendencies of the PCCRs and SCCRs are quite similar, we only illustrate the PCCRs in Figure 1A (see Supplementary Material) without a detailed description.

#### Frequency Distribution of Subskill Mastery Probability

**Figure 3:**
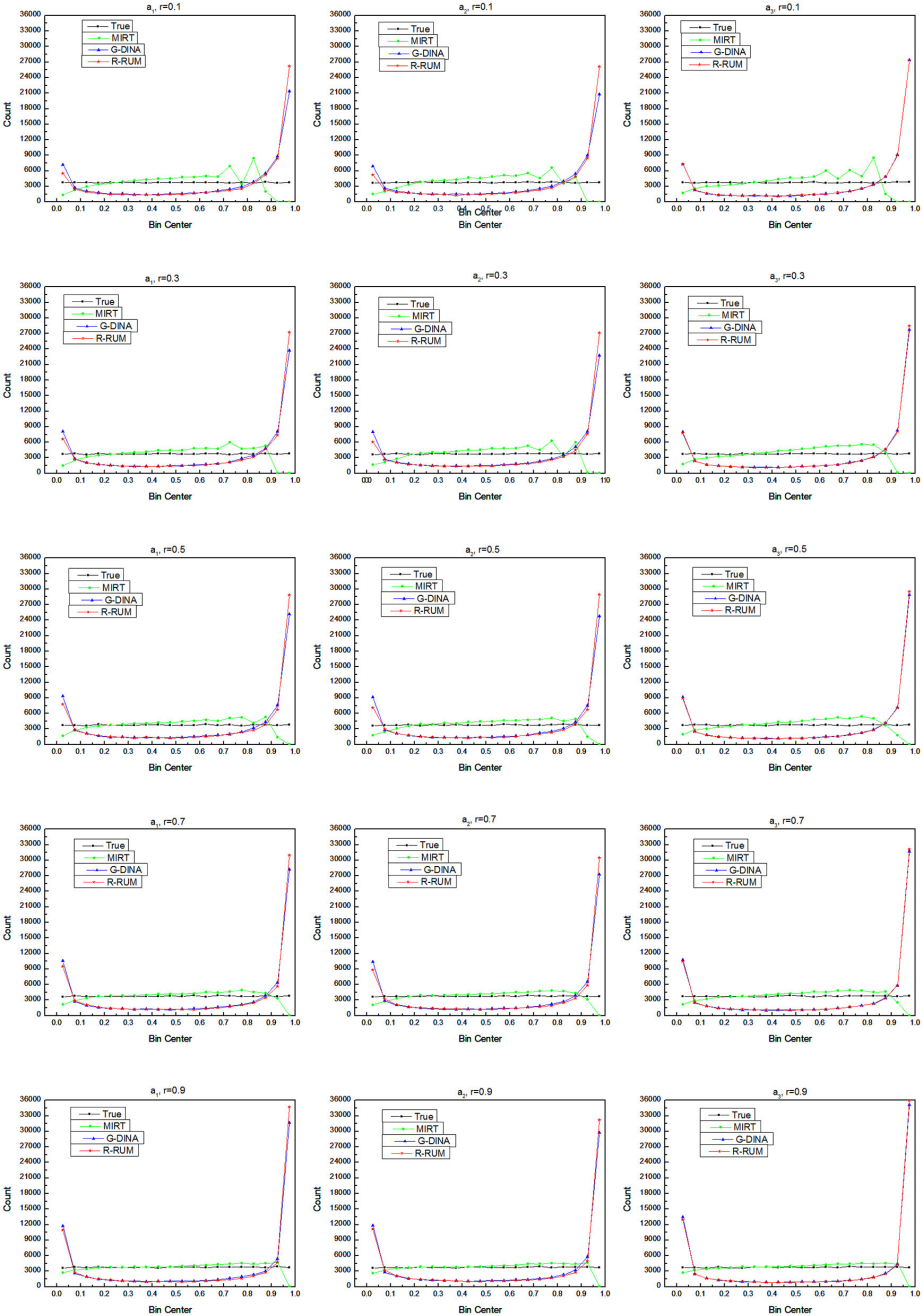
Comparison of frequency distributions of subskill mastery probability.

A comparison between the estimated frequency distributions and the true distribution can show the deviation of the estimated subskill mastery probabilities from the true values in the general value distribution. Figure 3 shows the frequency counts of the estimated subskill mastery probability vs. the true values, with the subskills mastery probabilities on the *x*-axis and the frequency counts on the *y*-axis.

The frequency distribution of the true value is almost a straight line under any simulation condition. The overall frequency distributions of the estimated subskill mastery probability for MIRT are close to their corresponding true values, with bias at the highest and lowest parts, and the bias decreases as the correlation of the subskills increases. The frequency distributions of the estimated subskill mastery probability for R-RUM and G-DINA are similar in shape, which is in line with the U-shaped distribution of Lee and Sawaki (2009b), with overwhelming counts at the highest and lowest ends and very low counts in the wide range of the middle of the *x*-axis; however, they are somewhat different from the true values. Compared to the true values, R-RUM and G-DINA underestimate a large number of average-scoring and low-scoring test-takers and overestimate many high-scoring test-takers. In addition, the frequency distribution obtained with R-RUM and G-DINA also reminds us that the overall frequency distributions obtained from dichotomous scale models will be U-shaped even if the true values are normally distributed and the corresponding true frequency distribution is a straight line.

## Discussion

The MIRT model, which hypothesizes that the latent variables are continuous, has a theoretical advantage in diagnostic analyses of reading comprehension tests. In this research, empirical and simulation studies were conducted to explore whether the model with a theoretical advantage has a practical advantage in reading diagnoses, and the findings demonstrated the practical advantage of MIRT in several ways. First, MIRT and two representatives of the traditionally widely used dichotomous scale models (R-RUM and G-DINA) were compared on model-data fit indices in the empirical analysis, and both the assessments of relative model-data fit (i.e., the −2 × log-likelihood, AIC, and BIC) and absolute model-data fit (i.e., *M*_2_ and the RMSEA) revealed that MIRT should be the chosen model. Second, a comparison of the relations between the estimated subskill scores from the three models and the true values indicated that the estimated subskill scores from R-RUM and G-DINA could not represent the true subskill abilities of test-takers as well as the estimated scores from MIRT. Third, the correct classification rates for the three models were explored under different research conditions in the simulation section, with the result that MIRT achieved the highest PCCRs and SCCRs under all conditions, and its improvement over the other two models increased as the subskill correlations increased. Finally, the estimated frequency distributions of the subskill mastery probability were compared to the true distribution, and the results revealed that the estimated frequency distributions of the subskill mastery probability from MIRT were more similar to the true values whereas, for R-RUM and G-DINA, the frequency distributions were very different from the corresponding true values. The wide variation between MIRT and the other two models in the frequency distribution of the subskill mastery probability matched their different hypotheses on latent variables, which also supported the inference that the continuous latent variable hypothesis benefits MIRT in gaining advantages in reading diagnostic analyses. These results were expected because the MIRT model treats the latent variables in question as continuous variables, which is in accordance with the nature of reading subskills; while R-RUM and G-DINA treat the latent variables in question as dichotomous variables, which would lead to information loss correspondingly (Buck and Tatsuoka, 1998; Bonifay and Cai, 2017).

Developing tests with a diagnostic purpose and retrofitting existing tests for diagnostic purposes are the two main methods presently available to obtain diagnostic information (DiBello et al., 2007), and there are pros and cons in both the types of methods. Developing tests with a diagnostic purpose will ultimately provide the user with more fine-grained diagnostic feedback, but the diagnostic reading test is barely in its infancy (Alderson et al., 2015) and few reading tests have been designed to provide diagnostic feedback until recently. Retrofitting existing tests for diagnostic purposes is convenient because the items and response data are already available, but it is certainly not the optimal method to obtain diagnostic information because the cognitive model being obtained in a *post-hoc* analysis depends on the existing test, and a sufficient number of subskills cannot be guaranteed due to the limited number of items in the existing test (Li et al., 2016). In contrast to the latter method, the former develops the diagnostic test with a well-articulated cognitive model from the beginning and can fully reflect the principles that underlie the cognitively diagnostic analysis, so developing tests with a diagnostic purpose is of critical importance to future reading diagnostic study (Jang, 2009). However, for now, retrofitting existing tests for diagnostic uses is currently a feasible approach to address the needs of diagnostic feedback from reading assessments, and almost all the published studies on reading diagnoses used the latter method (Buck et al., 1997; Jang, 2009; Lee and Sawaki, 2009b; Li et al., 2016). The real and simulated data of this study were based on an existing large-scale reading comprehension test, which was not designed to be diagnostic and was developed to measure the reading subskills being generally regarded as continuous latent variables (Griffin and Nix, 1991; Lumley, 1993; Grosjean, 2001; Smith, 2004). In short, similar to previous reading diagnostic studies, the reading diagnostic analysis in this study was conducted by retrofitting an already existing reading test to a diagnostic function. In practice, one of the central functions of reading diagnoses is to offer diagnostic information about the reading abilities of learners that may be used to guide teachers and learners in subsequent remedial teaching and learning. In this study, the empirical and simulation study results suggest that the MIRT model can produce more dependable diagnostic feedback, which will lead to more accurate reports about the weaknesses and strengths of the test-takers. A suitable model is a necessary prerequisite for a reading diagnosis to be useful. If traditional dichotomous scale models had been selected, our inferences about the reading abilities of far more test-takers would have deviated from the truth, and the remedial plans based on such information would be useless or misleading.

We should consider the dependability of feedback before the diagnosis can claim to be successful (Kunnan and Jang, 2009); however, to maintain the practical usefulness of a reading diagnostic analysis, the existence of dependable diagnostic information is not sufficient. The scores should also be understood and used properly; otherwise, even the best test is worthless (Brennan, 2006). In reading diagnoses, the score interpretation will be more reasonable with the MIRT model. With traditional dichotomous scale models, it may be difficult for teachers and students to properly understand the diagnostic results. As noted by Jang (2009), students may require a definition of “master” and should know that “master” cannot be interpreted as “no further action” in reading diagnoses. With dichotomous subskill scales, it may be difficult to explain why the state of “mastery” on a subskill is not equal to “flawless mastery.” With continuous subskill scales, however, we tell students that “mastery” means “strong ability,” which is related to scores that are higher than the required score but are not equal to “flawless mastery” because “mastery” implies an interval along a continuous scale rather than a point on a dichotomous scale.

In reading instruction, diagnosis can be regarded as the interface between learning and assessment (Alderson, 2005). Without dependable diagnostic information and reasonable score interpretation, however, it will be very difficult for a diagnosis to truly become such an interface (Kunnan and Jang, 2009; Alderson et al., 2015). In this regard, the MIRT model actually matters very much in improving the practical usage of the reading diagnosis.

Moreover, when being used in diagnostic practices, the MIRT model has other strengths, including more informative diagnostic information and being able to extend to model testlets. As a continuous subskill scale diagnostic model, in addition to the traditional diagnostic report of the mastery states of test-takers, MIRT is able to describe their locations on the continuous score scale of each subskill, which can provide the teachers and learners with more detailed diagnostic information. Another important advantage of MIRT is that it is easy to extend to fit data with testlets. Testlets commonly exist in current reading comprehension tests, which are presented as a group of items with the same stimulus developed and administered as a single unit (Wainer and Kiely, 1987). It has been observed that the bifactor model is successful in approximating response data on testlets (McLeod et al., 2001; DeMars, 2006), and the MIRT model is able to combine with the bifactor model to fit multidimensional data with testlets.

Nevertheless, we should know that the mastery states of test-takers from MIRT may sometimes be hard to obtain, because with MIRT, the two-step approach (estimating continuous θ values and then dichotomizing them with cut-off points) should be used to obtain these mastery states, and the establishment of a cut-off point tends to be labor-intensive and time-consuming (Hambleton and Pitoniak, 2006). For cases where the mastery state from MIRT becomes realistically infeasible, the mastery states from traditional diagnostic models in reading diagnoses can be regarded as a viable alternative to provide the diagnostic feedback under certain conditions, because though hypotheses on the latent variable are different, both MIRT and the traditional diagnostic models are actually popular multidimensional models that can be used for diagnostic purposes. These multidimensional models can help us to provide diagnostic information, while things would be difficult with the unidimensional model. Ma et al. (2020) recently showed that the traditional diagnostic models fitted the data from a cognitive diagnostic assessment better than the unidimensional IRT model, and they could extract useful diagnostic information while the general abilities estimated from the unidimensional model were of little diagnostic utility. Indeed, the diagnostic information previously obtained in reading diagnoses are mostly based on the traditional diagnostic models, and R-RUM and G-DINA are representatives of these models.

Regarding the comparison between R-RUM and G-DINA, G-DINA performed slightly better on the average PCCRs and SCCRs over the five simulation conditions; in addition, when used with empirical reading comprehension test data, G-DINA had slightly lower −2 × log-likelihood and AIC values and slightly higher BIC values, which coincides with the finding of Li et al. (2016) with the MELAB reading test. It should be noted, however, that the performance of R-RUM was only slightly worse than G-DINA for the most part, and in other circumstances, it had an edge over G-DINA, i.e., differences actually exist between these two models, but there is no huge dissimilarity, and in general, they perform similarly.

Considering that G-DINA is a saturated model, which would obtain a better model-data fit and correct classification rates (de la Torre, 2011), R-RUM actually performed well. Therefore, the R-RUM can be regarded as a good choice when there is a lack of diagnostic models with continuous scale, which also provides some support to previous reading diagnostic research with R-RUM, such as the studies by Jang (2009), Lee and Sawaki (2009b), and Jang et al. (2013).

Finally, the starting point of this study is the contradiction encountered in practice: statistical methods have theoretical assumptions, and typically, they should only be applied to data that meet their assumptions; however, in previous reading diagnostic studies, dichotomous scale models, whose hypothesis on latent variables are not in accordance with the continuous nature of reading subskills, have always been the chosen model. Therefore, the main focus of this study is to compare the performances of MIRT and the traditionally widely used dichotomous scale models when they are used in reading diagnostic analyses. All efforts in this study were oriented toward this practical requirement, including the criteria for selecting the representative models and calibration software programs: representatives of the traditionally widely used models should be widely recognized in previous studies, and the chosen software programs should be well-accredited and conveniently obtainable. For similar reasons, we simulated only datasets consisting of continuous latent variables in this study, with one continuous scale diagnostic model and two dichotomous scale diagnostic models being used to approximate them: using all approximating models as generating models simultaneously seems to be a more popular treatment; however, this study is focused on model selection for reading diagnoses instead of general model comparison, which means continuous latent variables are of particular interest to us, and the discussion about model performance on dichotomous latent variables has little relevance in the aim of this present study: even if R-RUM and G-DINA were used as generating models, the performances of the three models on datasets generated by these two models do nothing to aid in evaluating their abilities in reading diagnoses. In fact, when discussing model selection in reading diagnoses, we know that the theoretical assumption of MIRT on latent variables is in accordance with the continuous nature of reading subskills, and theoretically dichotomous scale models, such as R-RUM and G-DINA are at a disadvantage in the context of reading comprehension test data; so the aim of this present study is to compare the performances of models with the theoretical advantage to those of models traditionally chosen for reading diagnoses.

Similar to the issue that Buck and Tatsuoka (1998) raised more than 20 years ago, there is a longstanding contradiction in reading diagnostic studies: the subskills required by reading comprehension tests are generally regarded as continuous latent variables, whereas dichotomous scale models have been the traditional choice in previous reading diagnostic studies. This study focused on whether the MIRT model, a model with a continuous subskill scale and that can be used for diagnostic purposes, has advantages when used to diagnose reading comprehension tests. Based on comparisons on the model-fit statistics, relations between the estimated subskill scores and the true subskill score, correct classification rates, and frequency distributions of the subskill mastery probability, this study confirmed the MIRT model can produce more dependable diagnostic information, which means more accurate inferences about the weaknesses and strengths of test-takers and greater guidance value for remedial teaching and learning. In addition, with the MIRT model, the reading diagnosis is able to provide more informative diagnostic information and a more reasonable score interpretation and is convenient to extend to fit testlets. These are all important bases for the reading diagnosis to fulfill its practical function and to truly become the interface between learning and assessment. Therefore, the MIRT model warrants broad attention and is recommended for use in future reading diagnoses.

This study was conducted with real and simulated data based on the administration of a single reading comprehension test; therefore, only one set of test items and one Q-matrix was used. More conditions, such as different numbers and grain sizes of subskills (Harding et al., 2015) and different item qualities (being defined by item parameters), should be considered in future studies for a more comprehensive and detailed evaluation. In addition, only the commonly employed correct classification rates based on simulated data were used in the discussion of classification accuracies, while classification accuracies may be empirically examined through approaches used in the validation of diagnostic studies, such as interviews or controlled remediation of students (Tatsuoka, 2009). Future work is required to provide insights about the performances of these empirical approaches as well as the classification accuracies of competing models based on real data. Moreover, no polytomous scale model was chosen as representative of traditionally widely used models to compare with the MIRT model because scarce reading diagnoses with this category of diagnostic model have been conducted. Although a polytomous scale with more than two ability levels is considered to be a compromise between the continuous scale and the dichotomous scale (Fu, 2005), Haberman et al. (2008) have shown that polytomous scale models are competitive with MIRT when there are four or more ability levels for each subskill; thus, the performance of polytomous scale models in reading diagnoses awaits investigation.

Last but not the least, though subskill scores estimated based on a MIRT model are more accurate and reliable than the estimates based on many other subskill score estimation methods (Fu and Qu, 2018), previous study reveals that if the subskill scores are highly correlated, the subskill score may have no added-value (Sinharay, 2010). Therefore, in practical uses of subskill score estimation methods, we should first determine whether reporting subskill scores is reasonable, especially when the subskill scores are highly correlated.

## Data Availability Statement

The raw data supporting the conclusions of this article will be made available by the authors, without undue reservation.

## Author Contributions

HL and YB proposed the original thoughts, designed the study, contributed to the article, and approved the submitted version. HL completed the draft writing, with the support of YB. All authors contributed to the article and approved the submitted version.

## Conflict of Interest

The authors declare that the research was conducted in the absence of any commercial or financial relationships that could be construed as a potential conflict of interest.

## Publisher's Note

All claims expressed in this article are solely those of the authors and do not necessarily represent those of their affiliated organizations, or those of the publisher, the editors and the reviewers. Any product that may be evaluated in this article, or claim that may be made by its manufacturer, is not guaranteed or endorsed by the publisher.
