# Supplementary material for: Model Selection for Cogitative Diagnostic Analysis of the Reading Comprehension Test
Source: Front Psychol. 2021 Aug 13;12:644764. doi: 10.3389/fpsyg.2021.644764 (PMC8422035; doi:10.3389/fpsyg.2021.644764)

Appendix A: Brief introduction to R-RUM, G-DINA, and MIRT

The reduced reparametrized unified model (R-RUM)

The reduced reparametrized unified model (R-RUM) is written as follows:

$$P(X_{ni} = 1|\alpha_n; \pi^*, r_{ik}^*) = \pi^* \prod_{k=1}^{K} r_{ik}^{*(1-\alpha_{nk})\times q_{ik}}, \qquad (1)$$

where *P* is the probability of examinee *n* correctly answering item *i*; $\boldsymbol{\alpha}_n = (\alpha_{n1}, \dots, \alpha_{nk})$ represents whether examinee *n* masters each subskill or not, where $\alpha_{nk} = 0/1$ for each subskill; π^* is the probability of a correct answer to item *i* given all required subskills are mastered; r_{ik}^* is given by $\frac{P(Y_{nik}=1|\alpha_{ik}=0)}{P(Y_{nik}=1|\alpha_{ik}=1)}$, and the farther r_{ik}^* is from 1, the better item *i* discriminates the masters and non-masters of the subskil *k*.

In addition, R-RUM is a variant of RUM. The RUM is also termed as the fusion model (FM), which is obtained by reparameterizing the unified model (Hartz, 2002). Usually it is sufficient to use R-RUM instead of RUM for practice application (DiBello & Stout, 2008).

The Generalized Deterministic Inputs, Noisy "and" Gate (G-DINA) model

Under the G-DINA model, examinees are classified into $2^{K_j^*}$ subskill patterns, where K_j^* represents the number of subskills required by item *j*. The G-DINA model is a saturated model, which can be expressed as follows:

$$P(\boldsymbol{\alpha}_{lj}^*) = \delta_{j0} + \sum_{k=1}^{K_j^*} \delta_{jk}\,\alpha_{lk} + \sum_{k'=k+1}^{K_j^*} \sum_{k=1}^{K_j^*-1} \delta_{jkk'}\alpha_{lk}\alpha_{lk'} \dots + \delta_{j12\dots K_j^*} \prod_{k=1}^{K_j^*} \alpha_{lk}, \qquad (2)$$

where *P* represents the probability of an examinee with subskill pattern $\boldsymbol{\alpha}_{lj}^*$ answering item *j* correctly; δ_0 represents the probability that an examinee with no required subskill responses correctly; δ_k, being a main effect, represents the change in the probability that an examinee with only subskill α_k leads; $\delta_{kk'}$, being a first-order interaction effect, represents the change in the probability that an examinee with both α_k and $\alpha_{k'}$ leads except δ_k and $\delta_{k'}$; $\delta_{12\dots K_j^*}$ represents the change in the probability that an examinee with all required subskills leads except main and lower-order interaction effects.

The Multidimensional Item Response Theory (MIRT) model

The form of the compensatory three-parameter logistic MIRT model is presented as

$$P(U_{ij} = 1|\boldsymbol{\theta}_j, \boldsymbol{a}_i, c_i, d_i) = c_i + (1 - c_i)\frac{\mathrm{e}^{\boldsymbol{a}_i\boldsymbol{\theta}_j' + d_i}}{1 + \mathrm{e}^{\boldsymbol{a}_i\boldsymbol{\theta}_j' + d_i}}, \qquad (3)$$

where *P* represents the probability of a correct response; $\boldsymbol{\theta}_j$ is an $1 \times \mathrm{m}$ vector representing the ability level of person *j* on each of the *m* dimensions, and $\mathbf{a_i}$ is a $1 \times \mathrm{m}$ discrimination vector with each element indicating the discrimination of item *i* on each dimension; c_i is the guessing parameter; d_i is the intercept term, which is a scalar (Reckase, 2009).

References

Hartz, S. (2002). *A Bayesian framework for the unified model for assessing cognitive abilities: Blending theory with practicality.* Unpublished doctoral dissertation, University of Illinois at Urbana-

Champaign.
DiBello, L., & Stout, W. (2008). *Arpeggio documentation and analyst manual* (Ver. 3.1.001) [Computer software]. St. Paul: MN: Assessment Systems Corporation.
Reckase, M.D. (2009). *Multidimensional item response theory*. New York: Springer.

Appendix B: Classification results

Table 1A SCCR$_1$s for R-RUM, G-DINA, and MIRT

cut-off	SC 1(r=0.1)			SC 2(r=0.3)			SC 3(r=0.5)			SC 4(r=0.7)			SC 5(r=0.9)		
point	R-RUM	G-DINA	MIRT	R-RUM	G-DINA	MIRT	R-RUM	G-DINA	MIRT	R-RUM	G-DINA	MIRT	R-RUM	G-DINA	MIRT
0.10	0.740	0.711	0.916	0.738	0.715	0.918	0.734	0.714	0.921	0.731	0.717	0.927	0.737	0.723	0.938
0.11	0.745	0.717	0.910	0.743	0.721	0.913	0.740	0.720	0.916	0.737	0.723	0.922	0.743	0.729	0.933
0.12	0.750	0.723	0.902	0.748	0.726	0.906	0.745	0.726	0.910	0.743	0.729	0.916	0.749	0.735	0.929
0.13	0.754	0.728	0.895	0.752	0.731	0.899	0.750	0.732	0.904	0.748	0.735	0.911	0.755	0.741	0.924
0.14	0.758	0.733	0.890	0.757	0.736	0.893	0.755	0.736	0.897	0.754	0.741	0.904	0.760	0.746	0.920
0.15	0.762	0.738	0.883	0.761	0.741	0.887	0.760	0.742	0.892	0.760	0.747	0.899	0.765	0.752	0.915
0.16	0.766	0.742	0.878	0.765	0.746	0.880	0.764	0.747	0.886	0.764	0.751	0.894	0.770	0.757	0.912
0.17	0.769	0.746	0.872	0.769	0.751	0.874	0.769	0.752	0.880	0.768	0.756	0.889	0.774	0.762	0.908
0.18	0.772	0.750	0.866	0.772	0.754	0.868	0.772	0.756	0.875	0.772	0.761	0.885	0.779	0.767	0.904
0.19	0.774	0.754	0.859	0.774	0.757	0.863	0.776	0.761	0.871	0.776	0.765	0.881	0.782	0.771	0.901
0.20	0.777	0.758	0.854	0.778	0.762	0.859	0.779	0.765	0.866	0.779	0.769	0.877	0.785	0.774	0.897
0.21	0.779	0.760	0.849	0.780	0.765	0.854	0.781	0.768	0.861	0.782	0.772	0.872	0.788	0.778	0.894
0.22	0.780	0.763	0.844	0.783	0.768	0.848	0.784	0.772	0.856	0.785	0.776	0.868	0.791	0.781	0.891
0.23	0.782	0.765	0.838	0.785	0.771	0.844	0.786	0.774	0.852	0.788	0.779	0.865	0.793	0.784	0.887
0.24	0.783	0.767	0.833	0.786	0.773	0.839	0.788	0.777	0.848	0.790	0.781	0.861	0.795	0.786	0.885
0.25	0.784	0.770	0.829	0.787	0.775	0.834	0.790	0.779	0.844	0.792	0.784	0.858	0.798	0.789	0.882
0.26	0.784	0.771	0.824	0.788	0.777	0.830	0.792	0.782	0.840	0.793	0.786	0.856	0.799	0.791	0.880
0.27	0.785	0.773	0.821	0.789	0.779	0.827	0.793	0.783	0.836	0.795	0.788	0.852	0.801	0.793	0.877
0.28	0.785	0.773	0.817	0.790	0.780	0.823	0.794	0.785	0.833	0.796	0.790	0.849	0.802	0.794	0.875
0.29	0.786	0.775	0.813	0.791	0.781	0.819	0.794	0.786	0.830	0.796	0.790	0.846	0.803	0.795	0.872
0.30	0.786	0.776	0.809	0.791	0.782	0.816	0.794	0.787	0.827	0.797	0.792	0.843	0.803	0.796	0.870

0.31	0.786	0.777	0.804	0.791	0.783	0.812	0.795	0.788	0.824	0.798	0.793	0.840	0.804	0.797	0.868
0.32	0.785	0.777	0.800	0.790	0.783	0.809	0.794	0.788	0.822	0.798	0.793	0.838	0.804	0.797	0.866
0.33	0.784	0.777	0.797	0.789	0.783	0.806	0.794	0.789	0.820	0.797	0.794	0.835	0.803	0.797	0.864
0.34	0.783	0.776	0.793	0.788	0.782	0.803	0.793	0.789	0.816	0.797	0.794	0.834	0.802	0.797	0.861
0.35	0.781	0.775	0.789	0.786	0.782	0.799	0.792	0.789	0.814	0.796	0.793	0.832	0.801	0.797	0.859
0.36	0.779	0.775	0.786	0.785	0.782	0.797	0.791	0.788	0.812	0.795	0.792	0.830	0.800	0.796	0.858
0.37	0.777	0.774	0.783	0.784	0.781	0.795	0.789	0.787	0.808	0.793	0.792	0.828	0.799	0.795	0.856
0.38	0.775	0.773	0.781	0.781	0.780	0.791	0.787	0.786	0.807	0.792	0.791	0.825	0.797	0.794	0.854
0.39	0.773	0.771	0.778	0.779	0.778	0.789	0.785	0.784	0.804	0.789	0.788	0.824	0.795	0.792	0.852
0.40	0.770	0.769	0.775	0.777	0.777	0.788	0.782	0.782	0.802	0.787	0.787	0.822	0.793	0.791	0.850
0.41	0.766	0.767	0.772	0.774	0.775	0.785	0.780	0.781	0.801	0.786	0.786	0.821	0.790	0.788	0.849
0.42	0.763	0.764	0.770	0.771	0.773	0.782	0.777	0.779	0.798	0.783	0.784	0.819	0.787	0.786	0.848
0.43	0.760	0.762	0.768	0.767	0.770	0.780	0.774	0.776	0.797	0.780	0.781	0.816	0.784	0.783	0.847
0.44	0.757	0.760	0.766	0.764	0.767	0.778	0.771	0.774	0.795	0.777	0.779	0.815	0.780	0.780	0.846
0.45	0.753	0.757	0.765	0.761	0.764	0.777	0.767	0.771	0.793	0.774	0.776	0.813	0.777	0.778	0.845
0.46	0.750	0.754	0.763	0.757	0.761	0.775	0.764	0.768	0.792	0.770	0.773	0.812	0.774	0.775	0.844
0.47	0.745	0.751	0.762	0.753	0.758	0.774	0.760	0.765	0.791	0.766	0.769	0.811	0.770	0.772	0.843
0.48	0.741	0.747	0.761	0.749	0.754	0.773	0.756	0.761	0.789	0.761	0.765	0.809	0.765	0.768	0.842
0.49	0.737	0.744	0.759	0.744	0.751	0.772	0.752	0.758	0.788	0.757	0.761	0.807	0.761	0.764	0.841
0.50	0.732	0.740	0.759	0.740	0.747	0.770	0.746	0.753	0.786	0.753	0.757	0.807	0.757	0.760	0.839
0.51	0.727	0.736	0.758	0.735	0.743	0.769	0.742	0.750	0.785	0.748	0.753	0.808	0.752	0.756	0.838
0.52	0.722	0.732	0.758	0.730	0.738	0.768	0.737	0.745	0.785	0.743	0.749	0.807	0.746	0.751	0.837
0.53	0.717	0.728	0.758	0.725	0.733	0.768	0.732	0.741	0.784	0.738	0.744	0.807	0.741	0.746	0.837
0.54	0.711	0.723	0.757	0.719	0.729	0.768	0.726	0.736	0.783	0.733	0.740	0.807	0.735	0.741	0.836
0.55	0.706	0.719	0.757	0.714	0.724	0.769	0.721	0.731	0.784	0.728	0.735	0.806	0.730	0.736	0.836
0.56	0.700	0.714	0.757	0.709	0.720	0.768	0.715	0.726	0.784	0.722	0.730	0.806	0.723	0.730	0.835

0.57	0.694	0.709	0.757	0.703	0.714	0.769	0.710	0.721	0.784	0.716	0.724	0.806	0.717	0.724	0.836
0.58	0.689	0.704	0.758	0.697	0.709	0.770	0.704	0.716	0.784	0.709	0.718	0.806	0.711	0.719	0.836
0.59	0.682	0.699	0.759	0.691	0.704	0.769	0.698	0.711	0.785	0.703	0.712	0.806	0.705	0.714	0.837
0.60	0.676	0.693	0.759	0.685	0.699	0.771	0.692	0.705	0.786	0.697	0.706	0.807	0.698	0.708	0.836
0.61	0.670	0.688	0.761	0.678	0.693	0.771	0.685	0.699	0.786	0.691	0.701	0.808	0.692	0.701	0.837
0.62	0.663	0.682	0.762	0.672	0.687	0.771	0.678	0.692	0.787	0.684	0.695	0.808	0.685	0.695	0.837
0.63	0.657	0.676	0.763	0.665	0.680	0.772	0.671	0.685	0.789	0.678	0.689	0.809	0.678	0.689	0.838
0.64	0.649	0.670	0.764	0.658	0.674	0.773	0.663	0.678	0.790	0.671	0.682	0.809	0.671	0.683	0.839
0.65	0.642	0.663	0.766	0.650	0.666	0.775	0.656	0.672	0.792	0.664	0.676	0.811	0.664	0.675	0.840
0.66	0.636	0.657	0.768	0.643	0.660	0.778	0.649	0.665	0.794	0.656	0.669	0.811	0.656	0.668	0.840
0.67	0.628	0.650	0.770	0.635	0.653	0.780	0.641	0.657	0.794	0.649	0.661	0.813	0.648	0.660	0.840
0.68	0.620	0.643	0.772	0.628	0.646	0.783	0.634	0.651	0.795	0.641	0.654	0.815	0.641	0.653	0.841
0.69	0.612	0.636	0.774	0.620	0.639	0.784	0.627	0.644	0.797	0.632	0.646	0.815	0.632	0.645	0.842
0.70	0.605	0.629	0.777	0.612	0.631	0.785	0.619	0.637	0.800	0.624	0.638	0.817	0.624	0.637	0.842
0.71	0.597	0.622	0.779	0.604	0.624	0.788	0.611	0.628	0.801	0.616	0.631	0.819	0.617	0.630	0.843
0.72	0.589	0.615	0.783	0.597	0.617	0.790	0.603	0.621	0.804	0.608	0.623	0.821	0.608	0.622	0.845
0.73	0.582	0.607	0.786	0.589	0.609	0.794	0.595	0.614	0.807	0.599	0.614	0.823	0.599	0.613	0.846
0.74	0.574	0.600	0.790	0.580	0.601	0.797	0.587	0.606	0.810	0.591	0.606	0.825	0.590	0.605	0.848
0.75	0.565	0.592	0.795	0.573	0.593	0.800	0.579	0.598	0.813	0.583	0.598	0.828	0.582	0.597	0.850
0.76	0.557	0.584	0.800	0.564	0.586	0.805	0.571	0.590	0.816	0.574	0.590	0.832	0.573	0.588	0.852
0.77	0.548	0.576	0.804	0.556	0.578	0.810	0.562	0.582	0.820	0.566	0.582	0.835	0.564	0.580	0.854
0.78	0.540	0.568	0.810	0.547	0.569	0.816	0.554	0.574	0.825	0.558	0.573	0.839	0.556	0.572	0.856
0.79	0.531	0.559	0.814	0.538	0.561	0.821	0.545	0.565	0.830	0.549	0.565	0.843	0.547	0.563	0.859
0.80	0.523	0.551	0.820	0.529	0.552	0.826	0.537	0.557	0.835	0.540	0.556	0.847	0.538	0.554	0.862
0.81	0.514	0.542	0.825	0.520	0.544	0.831	0.527	0.548	0.841	0.531	0.548	0.851	0.529	0.545	0.865
0.82	0.505	0.535	0.829	0.511	0.535	0.836	0.519	0.540	0.845	0.523	0.539	0.857	0.519	0.536	0.870

0.83	0.497	0.526	0.834	0.502	0.526	0.842	0.509	0.530	0.851	0.513	0.529	0.862	0.510	0.526	0.874
0.84	0.488	0.517	0.841	0.494	0.518	0.848	0.500	0.521	0.857	0.504	0.521	0.867	0.500	0.517	0.878
0.85	0.479	0.509	0.850	0.485	0.509	0.855	0.490	0.512	0.863	0.495	0.512	0.872	0.490	0.508	0.882
0.86	0.470	0.500	0.859	0.475	0.500	0.863	0.481	0.503	0.870	0.486	0.503	0.878	0.481	0.498	0.887
0.87	0.460	0.491	0.870	0.466	0.490	0.871	0.472	0.494	0.876	0.476	0.493	0.884	0.472	0.489	0.893
0.88	0.450	0.481	0.880	0.456	0.481	0.881	0.462	0.485	0.884	0.467	0.484	0.890	0.463	0.481	0.898
0.89	0.441	0.472	0.890	0.446	0.471	0.891	0.453	0.475	0.893	0.457	0.475	0.896	0.453	0.471	0.904
0.90	0.432	0.463	0.900	0.437	0.463	0.900	0.443	0.465	0.902	0.448	0.465	0.905	0.444	0.462	0.910
average	**0.682**	**0.688**	**0.807**	**0.688**	**0.692**	**0.815**	**0.692**	**0.697**	**0.826**	**0.696**	**0.700**	**0.842**	**0.699**	**0.702**	**0.865**

Table 2A Mean Proportion of Masters Estimated from R-RUM and G-DINA

SC	R-RUM			G-DINA		
	s1	s2	s3	s1	s2	s3
0.1	0.665	0.671	0.661	0.633	0.634	0.659
0.3	0.660	0.665	0.658	0.634	0.632	0.652
0.5	0.654	0.662	0.652	0.632	0.631	0.644
0.7	0.651	0.655	0.647	0.633	0.632	0.642
0.9	0.656	0.647	0.644	0.638	0.631	0.637
average	**0.657**	**0.660**	**0.652**	**0.634**	**0.632**	**0.647**

Figure 1A Comparison of PCCRs among R-RUM, G-DINA, and MIRT

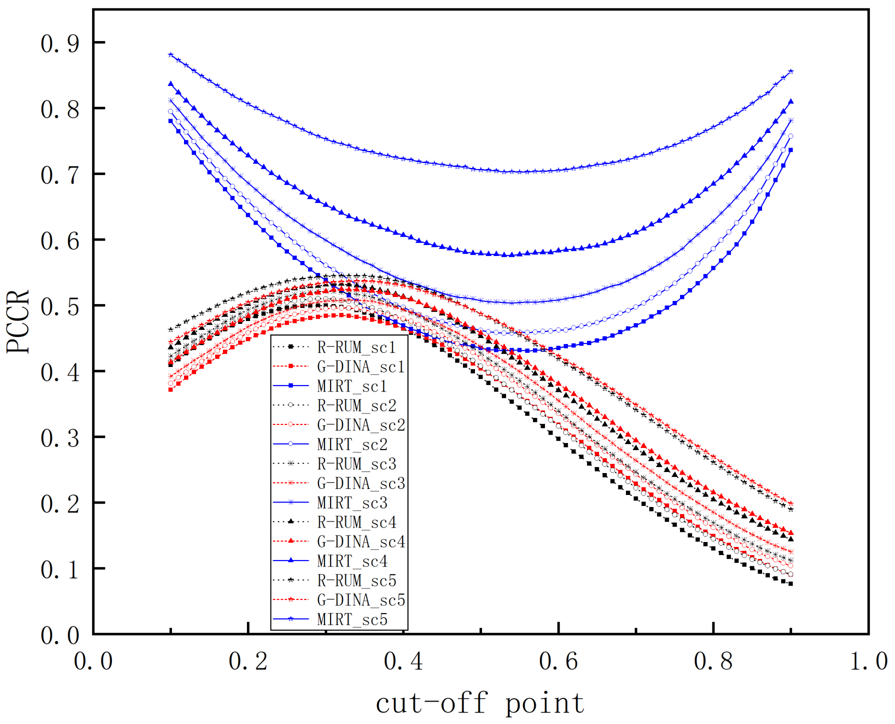

Supplement: Supplementary file 1 [file Data_Sheet_1.pdf]
